# Effects of intense pulsed light treatment on tear cytokines and clinical outcomes in meibomian gland dysfunction

**DOI:** 10.1371/journal.pone.0256533

**Published:** 2021-08-26

**Authors:** Qian Li, Junxiu Liu, Cheng Liu, Junfeng Piao, Wei Yang, Ningyu An, Jinyan Zhu

**Affiliations:** 1 Department of Ophthalmology, Ningxia Eye Hospital, Peoples’ Hospital of Ningxia Hui Autonomous Region, Ningxia Clinical Research Center on Disease of Blindness in Eye, First Affiliated Hospital of Northwest University for Nationalities, Yinchuan, Ningxia, China; 2 Medical Sci-Tech Research Center of Ningxia Medical University, Yinchuan, Ningxia, China; Boston University School of Medicine, UNITED STATES

## Abstract

Meibomian gland dysfunction (MGD) has become a prevalent ocular surface disorder. Its pathogenesis is regarded as a self-perpetuating inflammatory vicious circle. Intense Pulsed Light (IPL) treatment was recently applied to improve the meibomian gland function and reduce symptoms of MGD. However, studies investigating the change of specific inflammatory cytokines during IPL treatment remained sparse. To further figure out how IPL treatment modulates the inflammatory cytokines in tears of MGD, we therefore performed a cross-sectional study and enrolled 32 patients from March 2019 to December 2020. The patients received 3 sessions of IPL treatment (10 to 16 J/cm^2^) at 4-week interval. The signs and symptoms of MGD were evaluated by ocular surface disease index (OSDI), tear film breakup time (TBUT), and meibomian gland yield secretion score (MGYSS). The clinical evaluators and tear samples were analyzed at baseline and at each IPL treatment session. Concentrations of (chemokine ligand) CXCL1, (C-C motif chemokine) CCL11, (tumor necrosis factor) TNF-α, (interferon) IFN-γ, (interleukin) IL-2, IL-6 and (tissue inhibitor of metalloproteinase) TIMP-1were measured by Quantibody Human Dry Eye Disease Array1. OSDI significantly decreased after IPL treatment compared with baseline. TBUT and MGYSS increased consecutively during treatment. CXCL1, CCL11, TNF-α, IFN-γ, IL-2, IL-6 presented significantly decrease and TIMP-1 showed significantly increase from the pretreatment baseline. The changed concentrations of TNF-α, IFN-γ, IL-2, TIMP-1 correlated with TBUT, the changed values of CXCL1, TNF-α, IFN-γ, CCL11, IL-2, IL-6, TIMP-1 correlated with MGYSS, and the changed concentrations of CXCL1, IFN-γ, CCL11, IL-2, IL-6 correlated with TIMP-1. The data supported IPL treatment could significantly relieve both signs and symptoms of MGD. The therapeutic effect of IPL treatment may originate from regulation of inflammatory cytokines including CXCL1, TNF-α, IFN-γ, CCL11, IL-2, IL-6, and TIMP-1.

## Introduction

Dry eye disease (DED) represents a multifactorial disease of ocular surface characterized by a loss of homeostasis of tear film [1]. Because of failure to produce high quality or enough tears, DED can substantially cause ocular discomfort symptoms, affect vision, and impair patients’ quality of life [2]. DED has been categorized as aqueous deficiency and evaporative subtype [1]. Meibomian gland dysfunction (MGD) is a prevalent cause of evaporative DED, impacting more than 70% of individuals in some parts of the world, especially in Asian [3]. The pathogenesis of MGD is regarded as a self-perpetuating vicious circle: the chronic and diffuse inflammation of meibomian glands resulted in a stickier meibum production and terminal duct obstruction, the abnormal meibum deposited inside the meibomian glands and facilitated growth of bacterial, the subsequent release of toxic bacterial products and proinflammatory cytokines exacerbated inflammation of meibomian glands [4]. In addition, abnormal blood vessel growth surrounding the meibomian glands caused by chronic inflammation, in turn, produce and release more inflammatory mediators [3].

Treatments for MGD include topical antibiotic and anti-inflammatory agents, tear substitutes, warm compress and meibomian glands massage [5]. However, many MGD patients do not benefit from these treatments [6, 7].

Recently, intense pulsed light (IPL) has been applied as a novel therapeutic measure for MGD. IPL treatment could ameliorate symptoms and improve the condition of the tear film with relatively long-term effectiveness [8–11]. Despite the definite curative effect of IPL, the underlying mechanism of IPL remains unclear. There are several speculative theories about it. The light energy emitted by IPL device absorbed by hemoglobin, melanin, and water, transformed into heat causing destruction of blood vessels in the eyelid margin and adjacent conjunctiva. Therefore, preventing release of inflammatory cytokines from the meibomian glands and eyelids [12, 13]. IPL could also heat and liquefy the meibum, enable meibum to be secreted easily from terminal duct [14, 15]. Furthermore, the ability of IPL to induce coagulation and necrosis of *Demodex* [16], decrease proinflammatory cytokines in tears [17, 18] plays a key role in treating MGD.

However, studies investigating the change of specific inflammatory cytokines during IPL treatment remain sparse. To further figure out how IPL treatment interferes the inflammatory vicious circle of MGD, we analyzed tear cytokines including (chemokine ligand) CXCL1, (C-C motif chemokine) CCL11, (tumor necrosis factor) TNF-α, (interferon) IFN-γ, (interleukin) IL-2, IL-6 and (tissue inhibitor of metalloproteinase) TIMP-1. In addition, the clinical correlations with these cytokine levels were also analyzed.

## Materials and methods

### Patients selection

Patients were enrolled from the outpatient department of The Hospital of Ningxia Hui Autonomous Region from March 2019 to December 2020. The cross-sectional study adhered to the tenets of the Declaration of Helsinki and was approved by the ethics committee of the hospital. Written informed consent was obtained from all patients. Authors had access to information that could identify individual participants during data collection. Patients over 18 years of age and met diagnostic criteria of MGD [19, 20] were recruited in the study. The eye with a higher stage of MGD was enrolled in this study. Right eye was chosen once the MGD condition was equivalent in the two eyes. Informed consents were obtained from all patients. Patients were excluded if they met the criteria as follows: (1) pregnancy or lactation, (2) with autoimmune diseases, (3) with any ocular anatomical abnormality, (4) with any ocular infection or allergy, (5) with ocular surgical history or trauma within 6 months, (6) currently received any form of treatment for DED, (7) Fitzpatrick Skin Types IV, V and VI [21], (8) with pigmental lesions, skin cancer or tattoos in the ocular region.

### IPL treatment

Patients received 3 sessions of IPL treatment with the M22 system (Lumenis, Israel) at 4-week interval. The treatment was performed by a same doctor. The pulse intensity raged from 10 to 16 J/cm^2^ depending on the Fitzpatric skin type [21], pulse width was 6ms. The patients were asked to remove make-up and clean the treatment area on upper and lower eyelids. Ophthalmic proparacaine hydrochloride eyedrops were used 5 minutes before treatment. The ultrasound gel was applied to ocular region and mid face. A metal shields was placed in conjunctival sac to protect cornea and sclera, and the other eye was coved by an eyeshade during treatment. IPL flashes were placed for both upper and lower eyelid starting from the inner canthus and ending in the temporal region, with slight overlapping applications (4mm × 8mm for upper eyelid, 8mm × 15mm for lower eyelid, respectively). 2 passes of IPL were administered for each eye in order to ensure fully coverage of the treatment area. The patients received approximately 9 overlapping pulse for upper eyelid and 18 overlapping pulse for lower eyelid on each pass. Meibomian glands expression was performed for both eyelids using meibum expressor forceps after IPL treatment. The patients were instructed to use sodium hyaluronate eyedrops during the follow-up.

### Clinical evaluation

The clinical evaluations were performed at baseline and at each IPL treatment session. All measurements were conducted prior to IPL treatment. Subjective symptoms were estimated by the ocular surface disease index (OSDI). Tear film break-up time (TBUT) was facilitated by observing with a blue filter after applying fluorescein-strip into the inferior conjunctival sac. TBUT was measured 3 times for each patient and average was calculated. Meibomian gland yield secretion score (MGYSS) was used to scored meibum quality (0: clear and fluid-like, 1: cloudy and fluid-like, 2: cloudy and granular, and 3: whitish, toothpaste-like) [22]. The score was recorded according to the number of five glands where a meibum secretion could be expressed.

### Tear sample collection and cytokine analysis

Tear samples were collected before any other operations. The samples were collected after instillation of 60μL of phosphate-buffered saline into the inferior conjunctival sac. The patients moved eyeballs to mix the tear fluid content before sampling [23]. The samples were stored in EP tube and kept cold (4°C) during collection, and then stored at -80°C until further analysis. The concentrations of tear cytokines were detected following a protocol of Quantibody Human Dry Eye Disease Array1 (RayBiotc Inc, Norcross, USA).

### Statistical analysis

Data were expressed as mean ± standard error of the mean (SEM). The data of tear cytokines were also transformed into ratios by using the baseline value as the reference. Shapiro–Wilk test with SPSS 26.0 for Windows Software (SPSS Inc., Armonk, NY) was used to test the normality of data. A Paired Samples T Test was used to compare the baseline and posttreatment cytokine concentration after each IPL treatment session. Correlations between the tear cytokines and clinical parameters, and between their changed values after IPL treatment were analyzed by Spearman correlation coefficient, respectively. *P* < 0.05 was statistically significant.

## Results

### Clinical outcomes

**Fig 1:**
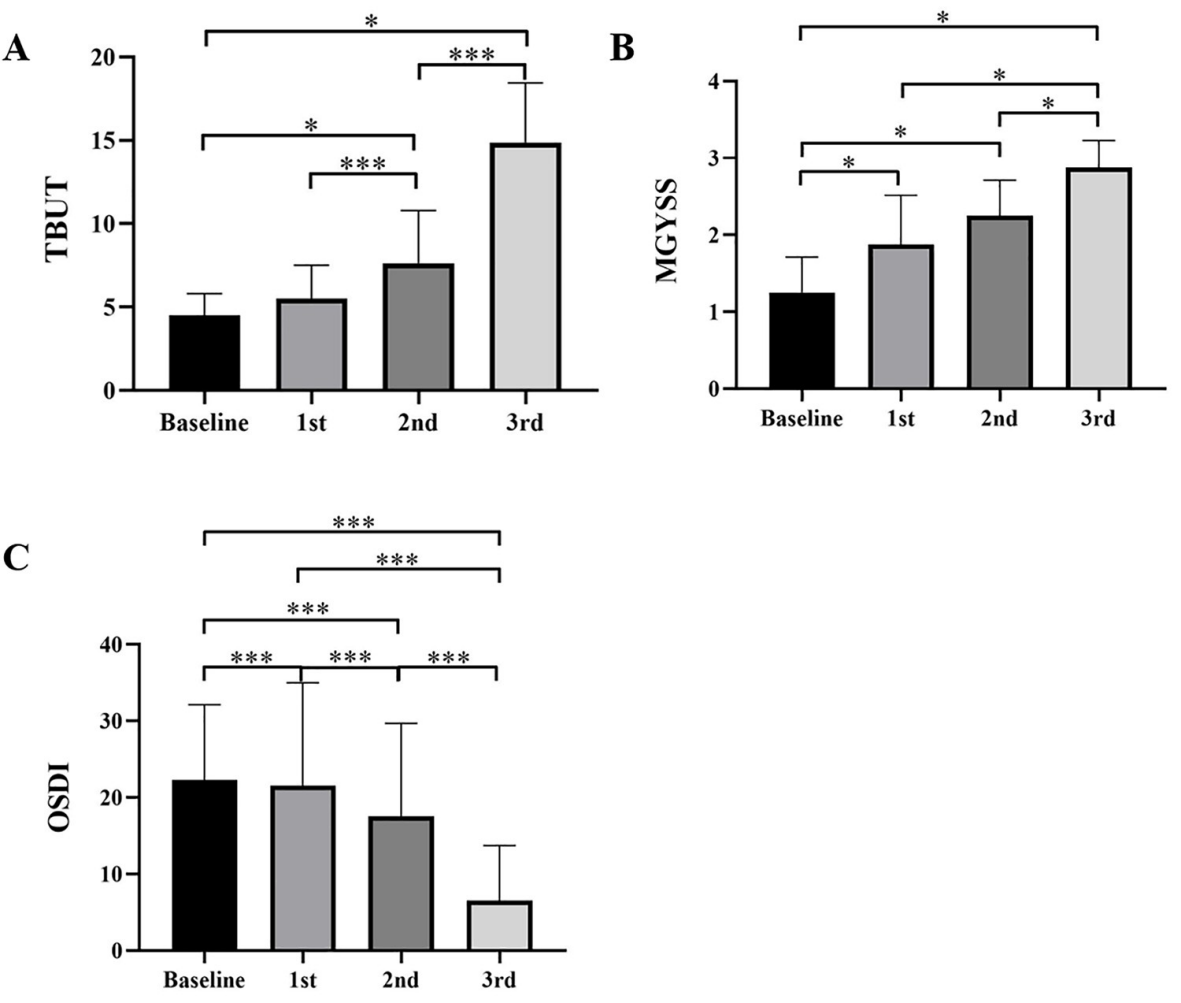
Change of clinical evaluators following each IPL treatment session in patients with MGD. (A) TBUT, (B) MGYSS, (C) OSDI. *P<0.05, ***P<0.001.

32 eyes of 32 patients (24 female and 8 male) were included into the study, the visual acuity and intraocular pressure of patients kept stable during IPL treatment. The mean (±SD) age of all the patients was 54.13±8.74 years (range, 43±65 years). OSDI decreased after IPL treatment compared with baseline, which were of statistically difference. TBUT and MGYSS increased consecutively during treatment. The clinical evaluators were listed in Table 1. Fig 1 showed the change of TBUT, MGYSS and OSDI following each IPL treatment session in patients with MGD.

**Table 1:** Clinical evaluators following each IPL treatment session in patients with MGD.

	Baseline	1st	2nd	3rd
OSDI	22.25±9.24	21.50±12.60	17.50±11.39	6.50±6.76
TBUT (s)	4.50±1.22	5.50±1.87	7.63±2.96	14.88±3.33
MGYSS	1.25±0.43	1.88±0.6	2.25±0.43	2.88±0.33

OSDI: ocular surface disease index.

TBUT: tear film break-up time.

MGYSS: meibomian gland yield secretion score.

### Change of tear cytokines

**Fig 2:**
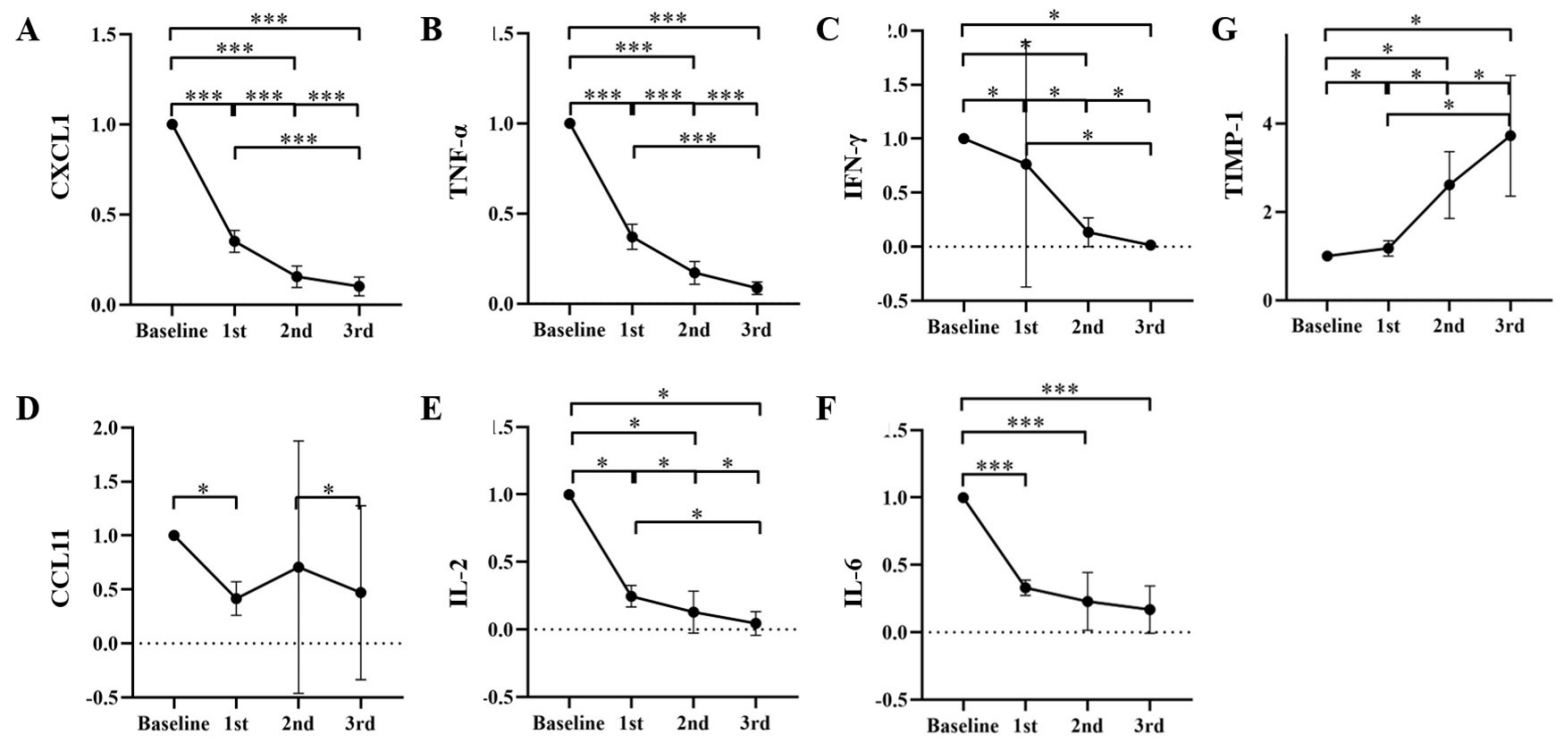
Change in tear cytokines following each IPL treatment session as a ratio compared with the pretreatment baseline. (A) CXCL1, (B) TNF-α, (C) IFN-γ, (D) CCL11, (E) IL-2, (F) IL-6, (G) TIMP-1. *P<0.05, ***P<0.001.

In the study we used a highly sensitive antibody microarray approach for the quantification of cytokines in tears of patients with MGD. The concentrations of CXCL1, TNF-α, IFN-γ, CCL11, IL-2, IL-6 presented significantly decrease and TIMP-1 showed significantly increase from the pretreatment baseline. CCL11 slightly raised after the second IPL treatment, however, the difference was not statistically significant (*P* = 0.07). The concentration of tear cytokines mentioned above was showed in Table 2. The change of tear cytokines including CXCL1, TNF-α, IFN-γ, CCL11, IL-2, IL-6 and TIMP-1 following each IPL treatment session as a ratio compared with the pretreatment baseline were presented in Fig 2.

**Table 2:** Concentration of tear cytokines following each IPL treatment session.

	Baseline	1st	2nd	3rd
CXCL1	3743.63±1079.63	1318.41±446.92	586.05±273.81	402.41±241.54
TNF-α	263.65±82.43	96.02±30.88	46.83±21.15	23.55±11.75
IFN-γ	123.36±59.77	45.46±18.67	10.76±4.40	1.76±1.31
CCL11	17.08±8.19	6.75±4.40	8.73±12.81	5.82±8.81
IL-2	16.02±7.51	4.07±2.73	1.84±1.65	0.55±0.76
IL-6	81.79±27.89	27.06±11.43	21.29±18.43	15.13±14.84
TIMP-1	5983.90+1726.65	7091.21+2496.08	15111.07+3907.47	21195.98+5645.63

### Correlation between clinical evaluators and tear cytokines

A correlation between the improvement of TBUT and decrease of TNF-α, IFN-γ, IL-2 after the entire IPL treatment was observed (*R* = -0.814, *P = 0*.*014; R* = -0.692, *P = 0*.*047; R* = -0.840, *P = 0*.*009*, respectively). The changed concentrations of CXCL1, TNF-α, IFN-γ, CCL11, IL-2, IL-6 after the entire IPL treatment correlated well with the increase of MGYSS (*R* = 0.845, *P = 0*.*008; R* = 0.764, *P = 0*.*027; R* = 0.764, *P = 0*.*027; R* = 0.732, *P = 0*.*039; R* = 0.845, *P = 0*.*008; R* = 0.845, *P = 0*.*008*, *respectively*). The correlations between the changed concentrations of CXCL1, IFN-γ, CCL11, IL-2, IL-6 after the entire IPL treatment and decrease of OSDI showed statistically significance (*R* = -0.172, *P = 0*.*038; R* = -0.757, *P = 0*.*030; R* = -0.713, *P = 0*.*047; R* = -0.868, *P = 0*.*005; R* = -0.794, *P = 0*.*019*, respectively). After the whole IPL treatment, the correlation analysis between the changed values of TIMP-1 and the changed values of TBUT, MGYSS and OSDI also showed statistically significance (*R* = 0.809, *P = 0*.*015; R* = -0.845, *P = 0*.*008; R* = -0.797, *P = 0*.*018*, respectively).

## Discussion

MGD is an increasingly prevalent ocular surface disorder and is one of the primary reasons for patient visits to ophthalmologist. Most available managements currently used are palliative solutions that often difficult to achieve long-term therapeutic effect. IPL treatment was recently introduced in the field of ophthalmology. Previous studies have confirmed the evident effect of IPL treatment in improving the meibomian gland function and reducing symptoms of MGD [10, 24, 25]. However, the exact mechanisms of IPL are still not fully elucidated.

The progression of MGD originates from imbalances between proinflammatory cytokines and protective immunoregulators of ocular surface [26]. Modulation of inflammatory cytokines in tears has been proposed as a possible reason for IPL in treating MGD [27].

Our study showed that the levels of CXCL1, CCL11 were significantly decreased in tears from patients with MGD. CXCL1 is a small peptide belonging to the CXC chemokine family that acts as a chemoattractant for several immune cells, especially neutrophils [28], and plays an important role in regulation of immune and inflammatory responses [29]. CCL11 is a chemokine originally implicated in the selective recruitment of eosinophils into inflammatory sites during allergic reaction [30]. Few studies found that CCL11 was involved in the pathogenesis of Sjögren’s Syndrome. CCL11, IFN-γ, and B-cell activating factor (BAFF) modulate Sjögren’s Syndrome pathology in a synergistic manner [31]. Serum levels of CCL11 evaluated in patients with Sjögren’s Syndrome and there was a link between CCL11 and disease activity [32]. Yet there were scarcely any studies about function of CXCL1 and CCL11 in MGD. One of the meaningful findings coming out of our study was that CXCL1 significantly decreased following each IPL treatment session. And the levels of CCL11 were found to be reduced compared with the pretreatment baseline, even though the values after second and third treatment were not statistically different. This may be because the reduction of CCL11 was not large enough and the sample size was too small. Further research is needed to determine by which CXCL1 and CCL11 levels are reduced during IPL treatment.

Furthermore, we observed a positive correlation between the reduction of OSDI and the change of CXCL1. OSDI elaborates dry eye symptoms including painful, gritty, blurred vision et, al. Accordingly, we speculate that the relief of symptoms, especially ocular pain, perhaps related to the decline of CXCL1. CXCL1 modulates pathological pain by stimulating the release of sympathetic amines and prostaglandins within the peripheral inflammatory sites [33]. Besides, CXCL1 indirectly drives peripheral nociceptive sensitization through its chemotactic effect mediating neutrophils migration [34].

IL-6 and IFN-γ has been proposed as biomarkers for DED. IL-6 and IFN-γ is secreted in the wake of desiccation stress on ocular surface [35, 36]. Across multiple studies, IL-6 was found to be raised in MGD patients [37, 38], and IL-6 levels have a negative relationship with Schirmer’s test score and TBUT [39], and a positive relationship with the ocular surface disease index (OSDI) score [40]. IFN-γ levels correlate very well with tear osmolarity, ocular surface staining, and Schirmer’s test scores [36, 41]. IFN-γ could induce loss of conjunctival goblet cell, thus decreases mucin production on ocular surface, worse the hyperosmolarity of tears [36, 42].

TNF-α could reduce tear production and stimulate matrix metalloproteinase (MMP) activation and limit fibrosis [43, 44]. Several reports have pointed out TNF-α raised significantly in DED patients, and the change of TNF-α correlated with disease severity, OSDI and Schirmer’s test score [39, 40, 44]. TNF-α and IFN-γ have been showed to induce corneal epithelial barrier dysfunction, which is a key feature of dry eye, through downregulation of the corneal epithelial cell adhesion molecules of tight junctions [45, 46].

In our study, IL-6, IFN-γ and TNF-α decreased significantly following IPL treatment in MGD patients, and the decrease of these cytokines was similarly correlated strongly with the improvement of TBUT, MGYSS and OSDI. These findings consistent with that of previous studies, demonstrated that IPL treatment enabled to significantly downregulated inflammation which is the core mechanism of MGD. The possible reason may be that IPL selectively ablates the superficial vessels located in eyelid margin, prevents the continued secreting of cytokines. In addition, the thermal interaction of IPL may also make the meibum less viscous, thus facilitate meibomian gland expression and unclogging of the gland. And last, the natural tendency of the eyelids to lose rigidity and elasticity may be reversed by the photomodulation of IPL, which assisted in returning eyelids to normal position and accomplishing complete blinks [47]. The process ultimately increased meibum secretion and reduced tear evaporation.

Interestingly, we observed significant elevation of TIMP-1 in the current study. More importantly, the improvement of TBUT, MGYSS and OSDI was significantly correlated with the increase of TIMP-1. TIMP-1 is a natural collagenase inhibitor of MMP9, and has recently emerged as a decisive factor in several human pathologies [48]. MMP-9 overexpression has been found in in vitro and in vivo models of dry eye disease [49]. The increase in MMP-9/TIMP-1 in Sjögren’s syndrome patients’ saliva is strongly involved in destruction of glandular and salivary duct tissues [49]. According to our data and previous studies, we speculated that TIMP-1 is involved in the disease process of MGD. Further studies are still needed to prove the hypothesis.

There are several limitations to our study. First, the study design was observational case series and lack of a control group. Second, the sample size was small and the duration of follow-up was limited to 4 weeks after final IPL treatment. Third, the patients received IPL treatment immediately followed by meibomian glands expression, which may help to clear clogged meibomian glands duct. However, previous studies have confirmed the improvement of dry eye symptoms and signs after IPL treatment alone. Furthermore, randomized controlled studies with a larger number of patients and longer follow-up period are necessary to assess the long-term effectiveness and safety of IPL treatment. Meanwhile, more exploratory studies are required to elucidate the function and potential mechanism of different inflammatory cytokines in tears of MGD.

## Conclusion

In conclusion, the study supported the efficacy of IPL treatment in relieving both signs and symptoms of MGD. The therapeutic effect of IPL treatment may originate from regulation of inflammatory cytokines including CXCL1, TNF-α, IFN-γ, CCL11, IL-2, IL-6 and TIMP-1.
